# Evaluation and Management of Syncope: Comparing the Guidelines of the American College of Cardiology/American Heart Association/Heart Rhythm Society and the European Society of Cardiology

**DOI:** 10.19102/icrm.2018.091208

**Published:** 2018-12-15

**Authors:** Noah N. Williford, Chad C. Ward, Brian Olshansky

**Affiliations:** ^1^Department of Cardiology, University of Iowa, Iowa City, IA, USA; ^2^Department of Internal Medicine, University of Iowa, Iowa City, IA, USA

**Keywords:** Cardiac implantable electronic device, guidelines, syncope, tilt-table testing

## Introduction

Syncope, a form of transient loss of consciousness, is a common medical problem that cardiac electrophysiologists deal with nearly daily. Care for patients with syncope can be challenging and complex. The primary goals are to diagnose the etiology of the problem; determine the risk of adverse outcomes; reduce unnecessary evaluation; and prevent recurrent episodes and hospitalizations, debilitation, sudden cardiac death, and total mortality.

To help clarify the issues, recent guidelines from the American College of Cardiology/American Heart Association/Heart Rhythm Society (ACC/AHA/HRS)^1^ and from the European Society of Cardiology (ESC),^2^ respectively, offer guidance and a review of presently available data. While the documents concur on many issues, differences between them also exist. Here, we review critical discrepancies as well as a number of agreements between the guidelines.

## Diagnosis and testing

Regarding the initial evaluation, the two sets of guidelines agree that a detailed history and physical examination are critical and represent the cornerstone of the evaluation **(Table 1)**. However, the ACC/AHA/HRS give the history a class I recommendation, while the ESC gives no specific recommendation. Both emphasize orthostatic vital signs (often missed in many evaluations), and both consider prognostic implications to be critical.

Video recordings, including those from “smartphones,” are suggested as being “helpful when available” in the ACC/AHA/HRS guidelines. Conversely, the ESC puts more weight on home video-recordings of spontaneous events with a class IIa recommendation and video-recorded tilt-table testing with a IIb recommendation, respectively. The electrocardiogram is given a class I recommendation in the ACC/AHA/HRS guidelines, but no class is specified for such in the ESC guidelines.

Separately, both guidelines emphasize the importance of risk stratification **(Table 2)**. The ACC/AHA/HRS guidelines give a class I recommendation for short- and long-term risk assessment, while no class recommendation is specified in the ESC guidelines. The ACC/AHA/HRS guidelines additionally note that short-term risk (< 30 days) depends upon etiology and reversibility, while long-term risk depends on comorbidities and treatment efficacy. The ACC/AHA/HRS guidelines give risk stratification scoring a class IIb recommendation.

One difficult decision physicians face is whether to discharge a patient from the emergency department or admit them for evaluation. With regard to disposition **(Table 3)**, the ACC/AHA/HRS guidelines substratify based on the seriousness of the suspected underlying medical condition. The ESC guidelines suggest outpatient management if low-risk features are present (class I), while stating that admission and evaluation are warranted for high-risk features. Furthermore, the ESC guidelines state that medium-risk patients should be observed in the emergency department or in a specialized syncope unit.

Directed testing is critical to proper syncope evaluation; however, frequently, the wrong evaluation is performed. There are no strong recommendations regarding blood testing, computed tomography (CT)/magnetic resonance imaging (MRI), or transthoracic echocardiography in the ACC/AHA/HRS guidelines, which provide a class II recommendation dependent upon the underlying diagnostic suspicion. With regard to transthoracic echocardiography, the ACC/AHA/HRS guidelines give a IIa recommendation if structural heart disease is suspected, while the ESC guidelines provide a class I recommendation for the same patient group. The ACC/AHA/HRS guidelines additionally recommend against echocardiography in those with expected low-yield results **(Table 4)**.

With regard to CT/MRI, the ACC/AHA/HRS guidelines provide a class IIb recommendation; however, no specific recommendation is given in the ESC guidelines. With regard to stress testing, there is a class IIa recommendation in the ACC/AHA/HRS guidelines and a class I recommendation in the ESC guidelines, respectively. Importantly, both guidelines suggest stress testing only if syncope occurs during or shortly after exertion. The ACC/AHA guidelines caution about performing such testing in high-risk populations, such as those individuals with critical aortic stenosis. Coronary angiography is not discussed specifically with regard to syncope in the ACC/AHA/HRS guidelines but, in the ESC guidelines, it is given a class IIa recommendation (similar to that of nonsyncope patients).

Both documents recommend against the performance of an undirected battery of useless tests (unless there is a high level of suspicion) that tend to be negative and expensive [eg, head CT, electroencephalogram (EEG), Holter monitoring, carotid Doppler].

Importantly, cardiac monitoring is given a class I recommendation for continuous in-hospital monitoring in patients with suspected cardiac etiology **(Table 5)**. The ESC guidelines recommend the use of implantable loop recorders (ILRs) early in the evaluation of patients with recurrent syncope of uncertain origin in the absence of high-risk features. In contrast, the ACC/AHA/HRS guidelines offer a class IIa recommendation for external cardiac monitoring and implantable cardiac monitoring if an arrhythmic etiology is suspected. The ESC guidelines note that Holter monitors should be considered if syncope occurs more than once per week (class IIa indication), while ILRs are warranted in those with less frequent syncope.

The ACC/AHA/HRS guidelines give a class IIa recommendation for electrophysiology study (EPS) in patients with syncope of suspected arrhythmic etiology, but do not recommend EPS for those with a normal electrocardiogram and/or normal ventricular function and structure unless an arrhythmic etiology is suspected. The ESC guidelines give a class I recommendation for patients with previous myocardial infarction or scar or in cases where syncope remains unexplained after noninvasive evaluation and a class IIa recommendation for those with bifascicular block when syncope remains unexplained after noninvasive testing, respectively. The ESC guidelines additionally provide a stronger recommendation for EPS than the ACC/AHA/HRS guidelines do, though both suggest EPS when the etiology of syncope remains unclear despite noninvasive testing **(Table 6)**.

Both guidelines give a class IIa recommendation for tilt-table testing when reflex syncope is suspected. Both guidelines also consider the use of other autonomic testing under select circumstances. The ACC/AHA/HRS guidelines propose tilt-table testing if psychogenic pseudosyncope is suspected and to distinguish between convulsive and true syncope (class IIa recommendation).

The ACC/AHA/HRS guidelines do not recommend the use of tilt-table testing (class III) to predict response to treatment **(Table 7)**. While the ACC/AHA/HRS guidelines consider EEG and hemodynamic measures during select tilt-table testing (class IIa recommendation), MRI, CT scan of the head, EEG, and carotid artery imaging in the absence of focal neurological findings or trauma are eschewed (class III). The ESC guidelines concur on this point (ie, no use for EEG, ultrasound of the neck, CT scan, or MRI unless other indications are present). These tests are frequently performed and are of no value for the most part.

## Special patient populations and treatment

To manage specific cardiovascular conditions, both guidelines give similar recommendations regarding the use of pacemakers for sick sinus syndrome and symptomatic bradycardia regardless of whether for second- or third-degree atrioventricular block. A class I recommendation is given for these particular indications; references are made to other society guidelines documents. Both guidelines concur on a class IIa recommendation for pacemaker implantation when the indication is less clear: examples include a heart rate of less than 40 bpm, sick sinus syndrome poorly correlated with syncope, and prolonged sinus node recovery time. A pacemaker is not recommended for implantation in those in whom sinus node dysfunction is asymptomatic (ACC/AHA/HRS) or if there is a reversible cause for bradycardia (ESC).

The ACC/AHA/HRS and ESC give similar recommendations for supraventricular tachycardia management with regard to consideration for EPS and possible ablation (class I) and for the use of antiarrhythmic drugs (class IIa). In the case of atrial fibrillation, both the ESC and the ACC/AHA/HRS strongly recommend rate control or rhythm control (class I), but the ESC goes further and recommends pacing if the ventricular response is slow.

With regard to ventricular tachycardia (VT), if there is a history of remote myocardial infarction, EPS is recommended (class I) by both the ACC/AHA/HRS and the ESC. The ACC/AHA/HRS guidelines specifically describe issues with regard to wide complex tachycardia of uncertain origin and the use of implantable cardioverter-defibrillators (ICDs) in patients with syncope suspected to be due to ventricular arrhythmias. The ESC considers inducible VT ablation and ICD implantation in patients with syncope thought to be due to VT with a left ventricular ejection fraction of less than or equal to 35%. Similarly to the ACC/AHA/HRS guidelines, ICD implantation is indicated in patients with syncope and previous myocardial infarction who have VT induced during EPS. Additionally, the ESC guidelines recommend management of VT by current ESC guidelines for patients with no structural heart disease and syncope. Similar to the ACC/AHA/HRS guidelines, ICD implantation is indicated in patients with syncope and previous myocardial infarction who have VT induced during EPS.

EPS, for risk stratification, has a class IIa recommendation in the ACC/AHA/HRS guidelines for those with a remote myocardial infarction, nonsustained VT, and left ventricular ejection fraction of less than or equal to 40%. The ESC guidelines give a class IIa indication for an ICD if the ejection fraction is more than 35% with recurrent syncope due to VT in cases where ablation cannot be performed and/or medication has failed.

ICD implantation based on other accepted criteria is recommended despite other suspected causes for syncope (class I) in both guidelines. The ACC/AHA/HRS guidelines are more specific, whereas the ESC guidelines allow for more physician discretion.

With regard to valvular heart disease thought to be the cause of syncope, a class I indication is given for aortic valve replacement with severe aortic stenosis in the ACC/AHA/HRS guidelines, while the ESC document does not give specific treatment recommendations regarding aortic valve replacement.

Considering patients with hypertrophic cardiomyopathy (HCM) leading to syncope, the ACC/AHA/HRS recommend an ICD if syncope is thought to be due to an arrhythmia, whereas the ESC recommends an ICD based on the HCM Risk-SCD score and the presence of provocable left ventricular outflow tract obstruction (both class I). The ESC gives a stronger recommendation with regards to exercise transthoracic echocardiography (class I), while the ACC/AHA/HRS give a class IIa recommendation. The ESC also suggests a class IIa recommendation for ILR use in patients at low risk but who have HCM.

With regard to arrhythmogenic right ventricular cardiomyopathy (ARVC), a class I recommendation for an ICD is given in the ACC/AHA/HRS guidelines for patients with syncope and documented sustained ventricular arrhythmias. A class IIa recommendation for ICD implantation is also given for those with unexplained or arrhythmic-appearing syncope, as this has been consistently associated with an increased risk of sudden cardiac death. Similarly, the ESC recommendations state that ICD can be considered (class IIb), but also include ILR as an option for those patients who are at low risk for sudden cardiac death (class IIa). Recommendations for an ICD (class I) for cardiac sarcoidosis with documented spontaneous sustained VT are given in the ACC/AHA/HRS guidelines, while cardiac sarcoidosis is not discussed in the ESC guidelines.

With regard to Brugada syndrome, both the ACC/AHA/HRS guidelines and the ESC guidelines recommend an ICD (class IIa) if syncope is unexplained or suspected to be of arrhythmic etiology. The ESC suggests an ICD for type I Brugada pattern and recommends consideration of an ILR in the lower-risk population, whereas the ACC/AHA/HRS document does not distinguish between patterns of Brugada syndrome and gives a class IIb recommendation for invasive EPS. The ACC/AHA/HRS do not recommend an ICD if syncope is reflex-mediated, even when a Brugada pattern exists. For short QT interval syndrome, due to the small number of patients with the disease, the recommendations are not strong in either set of guidelines. The ACC/AHA/HRS guidelines state that ICD implantation may be considered (class IIb) in patients with syncope suspected to be of arrhythmic etiology, while the ESC offers no recommendations. Meanwhile, for long QT syndrome, the use of β-blockers is recommended in all patients except, perhaps, those with long QT syndrome type 3 (ESC guidelines). Both guidelines give a class IIa recommendation for an ICD if β-blocker therapy is ineffective; left cardiac sympathetic denervation is also considered to be a reasonable option.

In patients with catecholaminergic polymorphic VT (CPVT), β-blockers and exercise restrictions are strongly recommended in the ACC/AHA/HRS guidelines (class I), as is the use of flecainide and verapamil when necessary (class IIa). No specific recommendations are given in the ESC guidelines.

With regard to early repolarization, the ACC/AHA/HRS guidelines recommend that EPS should not be performed in the absence of other indications and give a class IIb indication for ICD implantation in patients with suspected arrhythmic syncope. The ESC does not provide specific guidance for this population, citing a lack of studies.

The ACC/AHA/HRS guidelines have a long list of unusual conditions that can contribute to or cause syncope. The workup and management of vasovagal syncope (VVS) can be complicated. Both guidelines give detailed and distinct recommendations. For example, in the ACC/AHA/HRS guidelines, patient education is recommended (class I). For those with a sufficiently long prodromal period, physical countermeasures are given a class IIa recommendation. For those with recurrent episodes not prevented by the avoidance of triggers, midodrine may useful (class IIa recommendation). Orthostatic training and/or fludrocortisone may also be considered (class IIb recommendation). β-blockers can be considered reasonable in patients over the age of 42 years (class IIb). A conservative approach including additional salt and fluid intake, withdrawing or reducing medications that may cause hypotension, and even considering selective serotonin reuptake inhibitors or dual-chamber pacing with recurrent VVS and prolonged spontaneous pauses is warranted. In comparison, the ESC guidelines recommend utilizing the patient history to determine the probability of syncope being vasovagal (class I). It is also recommended (class I) that the patient be given an explanation of the diagnosis, reassurance, and suggestions about triggers. A class IIa recommendation is noted for isometric exercises and the modification/discontinuation of medications that may be responsible.

Cardiac pacing is given a class IIa recommendation for those with recurrent syncope and spontaneous documented asystolic pauses (lasting more than three seconds if symptomatic or more than six seconds if asymptomatic). A class IIb recommendation is given for tilt training and for fludrocortisone and midodrine. Furthermore, cardiac pacing may be considered for those with asystolic responses on the tilt-table test who are 40 years of age or older or who demonstrate adenosine-sensitive syncope. β-blockers are not recommended for the management of patients with VVS per the ESC guidelines (class III recommendation) but “might be reasonable” (class IIb) per the ACC/AHA/HRS guidelines. Cardiac pacing is not recommended when there is no documented cardioinhibitory reflex.

In patients with carotid sinus hypersensitivity, the ACC/AHA/HRS guidelines consider pacing to be reasonable (class IIa) if there is a cardioinhibitory or mixed response. A class IIb recommendation for the use of a dual-chamber pacemaker when pacing is needed is noted. In comparison, the ESC gives a class I recommendation regarding diagnosis (bradycardia or hypotension that reproduce spontaneous symptoms) and a class IIa recommendation regarding management (pacing should be considered in those aged older than 40 years with frequent, recurrent, unpredictable syncope). Pacing is not recommended in the absence of a cardioinhibitory reflex.

Both the ACC/AHA/HRS and the ESC guidelines contain similar nonpharmacologic recommendations regarding orthostatic hypotension, though the former’s guidelines are more specific. Acute water ingestion for temporary relief and fluid resuscitation is recommended by the ACC/AHA/HRS guidelines in the setting of acute dehydration (class I). The ESC guidelines are similar, and education and lifestyle measures are also recommended (class I). However, the ACC/AHA/HRS guidelines give specific recommendations, stating that acute water congestion should consist of at least 240 mL, and preferably more than 480 mL for maximal benefit, while also noting that the presence of glucose and salt in the water may lesson the effects if orthostatic hypotension is neurogenic in nature (though such may be helpful if dehydration is the etiology; class IIa).

For neurogenic orthostatic hypotension, medication recommendations are similar (eg, midodrine, fludrocortisone) except for the fact that the ACC/AHA/HRS guidelines recommend droxidopa (only available in the United States and the only drug that is approved by the Food and Drug Administration for symptomatic neurogenic orthostatic hypotension) as class IIa (similar to the other medications). The ESC guidelines in comparison make no mention of droxidopa (the drug is not available in Europe). The guidelines also differ when it comes to pyridostigmine and octreotide, which are in the ACC/AHA/HRS recommendations as class IIb but are not in the ESC recommendations at all. Both guidelines also suggest the use of compression garments (class IIa), while the ESC also suggests head-up-tilt sleeping (class IIa).

Specific pediatric syncope recommendations are made in the ACC/AHA/HRS guidelines regarding evaluation (class I), tilt-table testing if VVS is suspected (class IIa), and midodrine in patients not responding to lifestyle modifications (class IIa), whereas no recommendations are made in the ESC guidelines except for the mention of infantile reflex syncope attacks and breath-holding spells being potential causes of syncope in the pediatric population. Pediatric workup in the ESC guidelines is similar to that in adults. In the ACC/AHA/HRS guidelines, β-blockers are not recommended for pediatric syncope. Separately, for older patients, the ACC/AHA/HRS recommend a comprehensive approach to syncope involving a geriatrician (class IIa recommendation) and state that it is reasonable to consider syncope as a cause for nonaccidental falls in older adults. The ESC gives no specific recommendations.

Regarding athletes, recommendations are made in the ACC/AHA/HRS guidelines, but there are no specific recommendations in the ESC guidelines. The ACC/AHA/HRS give a class I recommendation for cardiovascular assessment prior to resuming competitive sports if syncope occurs. A class IIa recommendation is made for assessment by a specialist for athletes with high-risk features as well as for extended monitoring for athletes with unexplained syncope on exertion after the initial evaluation. Returning to competitive sports is not recommended prior to an evaluation being completed by a specialist in specific high-risk groups such as those with phenotype-positive HCM, CPVT, long QT syndrome type 1, or ARVC.

## Conclusion

In summary, both sets of guidelines are extensive, well-referenced, and carefully constructed. For the most part, the guidelines, while written in completely different styles, provide nearly the same recommendations for most clinical scenarios and conditions. Slight differences exist in recommendations regarding workup as well as in recommendations of medications and the utilization of specific therapies under very specific situations, but these documents concur for the most part. Therefore, as two completely independent groups arrived at very much the same conclusions based on the present state of knowledge on syncope, deviation from these guidelines is not recommended.

## Figures and Tables

**Table 1:** History and Physical Examination

ACC/AHA/HRS	ESC	Important Similarities	Important Differences		Other Notes
**Class I:** A detailed history and physical exam should be performed, with an attempt made to determine if cardiac versus neurological versus other	**No class recommendation given:** A detailed history and physical exam should be performed, with an attempt made to determine if vasovagal versus hypotensive versus cardiac	Both guidelines recommend obtaining orthostatic vital signs	•	ACC/AHA/HRS gives class I recommendation regarding ECG; ESC gives no recommendation	Initial examination is the cornerstone of the evaluation
			•	ACC/AHA/HRS state that smartphone recordings “may be helpful,” while ESC suggests using home video-recordings (class IIa) and video tilt-table testing (class IIb)

ACC: American College of Cardiology; AHA: American Heart Association; HRS: Heart Rhythm Society; ESC: European Society of Cardiology; ECG: electrocardiogram.

**Table 2:** Risk Stratification

ACC/AHA/HRS		ESC	Important Similarities	Important Differences		Other Notes
•	**Class I:** Evaluation of the cause and assessment for the short- and long-term morbidity and mortality risks of syncope are recommended	**No class recommendation given:** Risk stratification is recommended	Both recommend short-term risk assessment	•	ACC/AHA/HRS recommend long-term risk assessment at initial presentation	N/A
•	**Class IIb:** Use of risk stratification scores may be reasonable in the management of patients with syncope			•	ACC/AHA/HRS suggest considering risk stratification scores	

ACC: American College of Cardiology; AHA: American Heart Association; HRS: Heart Rhythm Society; ESC: European Society of Cardiology; N/A: not applicable.

**Table 3:** Disposition

ACC/AHA/HRS		ESC	Important Similarities	Important Differences	Other Notes
•	**Class I:** Hospital evaluation and treatment recommended if patient has a potentially relevant serious medical condition	**Class I:** Low-risk: discharge from ED; high-risk: admission and evaluation; medium risk: observe in ED or syncope unit instead of admission	Both guidelines rely on risk stratification	ACC/AHA gives recommendations based on seriousness of underlying illness, while ESC focuses on presenting features	N/A
•	**Class IIa:** Manage presumptive reflex-mediated syncope in outpatient setting; in intermediate-risk patients with unclear etiology, structured ED observation can reduce hospital admission (structured = expedited access to cardiology/testing)				
•	**Class IIb:** Manage selected patients with suspected cardiac syncope in outpatient setting				

ACC: American College of Cardiology; AHA: American Heart Association; HRS: Heart Rhythm Society; ESC: European Society of Cardiology; ED: emergency department; N/A: not applicable.

**Table 4:** Additional Testing

Test	ACC/AHA/HRS		ESC	Important Similarities	Important Differences	Other Notes
Blood testing	•	**Class IIa:** Targeted blood tests are reasonable	**No recommendations:** More evidence needed	N/A	ACC/AHA/HRS state that BNP may be useful if cardiac etiology is suspected	N/A
	•	**Class IIb:** Usefulness of BNP is uncertain; levels may be elevated in those with a cardiac cause				
TTE	•	**Class IIa:** Can be useful in selected patients if structural heart disease is suspected	**Class I:** Indicated if structural heart disease suspected	Both recommend if structural heart disease suspected	ESC gives stronger recommendations	Some advocate for TTE in all patients with syncope; however, ACC/AHA/HRS give a class III recommendation if the test is believed to be low-yield
CT/MRI	•	**Class IIb:** May be useful in selected patients when TTE inconclusive or when suspecting ARVC, sarcoidosis, pulmonary embolism, etc.	**No level of evidence given:** Suggested if cardiac cause suspected with nondiagnostic TTE	N/A	ACC/AHA/HRS give stronger recommendations	N/A
Stress testing	•	**Class IIa:** Can be useful if presyncope/syncope occurs during exertion	**Class I:** Indicated in patients who experience syncope during or shortly after exertion; when syncope due to second- or third-degree AV block is confirmed or when AV block develops during exercise, even without syncope; and when reflex syncope is confirmed in cases where syncope is reproduced immediately after exercise in the presence of hypotension	N/A	ESC gives stronger recommendations	N/A
Coronary angiography	•	**No recommendation given:** Not discussed	**Class IIa:** Same indications as nonsyncope patients	N/A	The ESC provides a recommendation, while the ACC/AHA/HRS do not	N/A

ACC: American College of Cardiology; AHA: American Heart Association; HRS: Heart Rhythm Society; ESC: European Society of Cardiology; BNP: brain natriuretic peptide; TTE: transthoracic echocardiogram; CT: computed tomography; MRI: magnetic resonance imaging; ARVC: arrhythmogenic right ventricular cardiomyopathy; N/A: not applicable; AV: atrioventricular.

**Table 5:** Cardiac Monitoring

ACC/AHA/HRS		ESC		Important Similarities		Important Differences	Other Notes
•	**Class I:** Continuous ECG is useful in hospitalized patients with suspected cardiac etiology; choice of cardiac monitor should be determined on the basis of the frequency and nature of syncope events	•	**Class I:** Immediate in-hospital monitoring is recommended in high-risk patients; ILR is indicated in early evaluation in patients with recurrent syncope of uncertain origin in the absence of high-risk features and a high likelihood of a recurrence within the battery life of the device; and ILR is indicated in patients with high-risk criteria in whom comprehensive evaluation did not reveal etiology and who do not have an indication for ICD implantation	•	Both strongly recommend continuous ECG when cardiac etiology is suspected	ESC recommends ILR be used earlier on in the evaluation	N/A
•	**Class IIa:** If arrhythmic etiology suspected, external cardiac monitoring can be useful; additionally, implanted cardiac monitoring can be useful in the ambulatory setting if arrhythmic etiology suspected	•	**Class IIa:** Holter monitoring should be considered in patients with frequent syncope (more than one episode per week); ILR should be considered in those with events occurring more than once per month; and ILR should be considered in patients with suspected or certain reflex syncope presenting with frequent or severe episodes	•	Both state that outpatient monitoring should be considered when the diagnosis is unclear		

ACC: American College of Cardiology; AHA: American Heart Association; HRS: Heart Rhythm Society; ESC: European Society of Cardiology; ECG: electrocardiogram; ILR: implantable loop recorder; N/A: not applicable.

**Table 6:** Electrophysiology Testing

ACC/AHA/HRS		ESC		Important Similarities	Important Differences	Other Notes
•	**Class IIa:** EPS can be useful for the evaluation of selected patients with syncope of suspected arrhythmic etiology	•	**Class I:** In patients with syncope and previous myocardial infarction/scar, EPS is indicated when syncope remains unexplained after noninvasive evaluation	Both suggest EPS when etiology is still not clear after noninvasive testing	ESC gives stronger recommendations in favor of EPS	Most data are old per the ACC/AHA/HRS, who state that yield is 50% if structural heart disease is present and 10% if not (highest in older patients with heart failure)
•	**Class III:** Not recommended for evaluation in those with normal ECG and normal ventricular structure and function unless an arrhythmic etiology is suspected	•	**Class IIa:** In patients with syncope and bifasicular block, EPS should be considered when syncope remains unexplained after noninvasive testing			

ACC: American College of Cardiology; AHA: American Heart Association; HRS: Heart Rhythm Society; ESC: European Society of Cardiology; EPS: electrophysiology study; ECG: electrocardiogram.

**Table 7:** Other Testing

Test	ACC/AHA/HRS		ESC	Important Similarities	Important Differences	Other Notes
Tilt-table testing	•	**Class IIa:** If diagnosis is unclear after initial evaluation, tilt-table testing can be useful if VVS suspected; can be useful for patients with syncope and suspected delayed hypotension when initial evaluation is nondiagnostic; is reasonable to distinguish convulsive syncope from true epilepsy; and reasonable to establish a diagnosis of pseudosyncope	**Class IIa:** Should be considered in patients with suspected reflex syncope, hypotension, POTS, or psychogenic pseudosyncope	Both state testing is reasonable if diagnosis is unclear	The ACC/AHA/HRS suggest tilt-table testing be performed if pseudosyncope is being entertained and for distinguishing between convulsive and true syncope	N/A
	•	**Class III:** Not recommended to predict response to VVS treatment				
Other testing	•	**Class IIa:** EEG and hemodynamics can be useful during tilt-table testing to differentiate syncope, pseudosyncope, and epilepsy	**Class III:** EEG, ultrasound of neck arteries, CT, and MRI not indicated in patients with syncope	Both suggest that these studies are not indicated unless focal deficits noted	N/A	N/A
	•	**Class III:** MRI, head CT, EEG, and carotid artery imaging are not recommended in routine evaluation in the absence of focal neurological findings or head injury				

ACC: American College of Cardiology; AHA: American Heart Association; HRS: Heart Rhythm Society; ESC: European Society of Cardiology; VVS: vasovagal syncope; POTS: postural orthostatic tachycardia syndrome; N/A: not applicable; EEG: electroencephalography; MRI: magnetic resonance imaging; CT: computed tomography.
